# Microbiota and Nutritional Changes Following a Low-FODMAP Diet in Patients with Ulcerative Colitis and Irritable Bowel Syndrome: A Randomized Controlled Trial

**DOI:** 10.3390/microorganisms14081643

**Published:** 2026-07-28

**Authors:** Viridiana Montsserrat Mendoza-Martínez, Karen Lorena de León-Barrera, Guillermo Meléndez-Mier, Marcela Esquivel-Velázquez, Alberto Piña-Escobedo, Jaime García-Mena, Juan Manuel Vélez-Ixta, Martha Alison Santoyo-Chávez, Jorge Luis De-León-Rendón, Alejandro González-Tapia, Nallely Bueno-Hernández

**Affiliations:** 1 Proteomics and Metabolomics Laboratory, Research Division, Hospital General de México “Dr. Eduardo Liceaga”, Mexico City 06720, Mexico; viridiana_2909@hotmail.com (V.M.M.-M.); nut.karendeleon@gmail.com (K.L.d.L.-B.); esquivel.marcela@gmail.com (M.E.-V.); santoyoalison@gmail.com (M.A.S.-C.); 2 Faculty of Public Health and Nutrition (FASPyN), Universidad Autónoma de Nuevo León, Monterrey 66455, Mexico; melendez651@gmail.com; 3 Departamento de Genética y Biología Molecular, Cinvestav, Av. Instituto Politécnico Nacional, Mexico City 07360, Mexico; apinae@cinvestav.mx (A.P.-E.); jgmena@cinvestav.mx (J.G.-M.); juan.velezixta@ucalgary.ca (J.M.V.-I.); alejandro_tapia@ciencias.unam.mx (A.G.-T.); 4 Department of Pediatrics, Cumming School of Medicine, University of Calgary, Calgary, AB T2N 4N1, Canada; 5 Inflammatory Bowel Disease Clinic, Coloproctology Service, Hospital General de México “Dr. Eduardo Liceaga”, Mexico City 06720, Mexico; r.jorgedeleon@hotmail.com

**Keywords:** ulcerative colitis, irritable bowel syndrome, FODMAP, nutrition, gastrointestinal microbiome

## Abstract

The low-FODMAP diet (LFD) reduces gastrointestinal symptoms, restricting poorly absorbed short-chain carbohydrates to reduce colonic fermentation. It has been proposed as a dietary strategy for irritable bowel syndrome (IBS) and ulcerative colitis (UC) patients. However, this diet could affect patients’ nutritional status and alter their gut microbiota. We aimed to evaluate the effects of a 10-week LFD on nutritional status, quality of life, and gut microbiota in patients with IBS, UC, and a control group without gastrointestinal symptoms. A randomized, single-blind clinical trial was conducted, including adults with IBS and UC, as well as a control group. Participants were randomly assigned to receive either an LFD or a standard diet (STD). Nutritional status, stool consistency, bowel habits, and quality of life were assessed, and stool samples were collected. Stool microbiota was profiled by 16S rRNA gene sequencing (V3) and analyzed for alpha diversity, beta diversity and differential abundance. Body composition was assessed by bioelectrical impedance. The follow-up period lasted 10 weeks (W0 to W10). Analyses were conducted in the per-protocol population. In total, 86 patients were included (IBS n = 40, UC n = 34, Control n = 12) with an average age of 40 ± 11 years. After adjustment for baseline values, no statistically significant differences in anthropometric or body-composition outcomes were observed between the LFD and STD groups. Similarly, there was a notable reduction in diarrhea frequency (53.3% vs. 20%, *p* = 0.058). Additionally, patients with IBS and UC exhibited similar microbiota profiles at W0. Among UC patients following the LFD, there was increased presence of BX12, CAG-1427, Holdemania, Evtepia, and Granulicatella, while Angelakisella and CAG-177 were less abundant. LFD was associated with changes in stool consistency, bowel habits, quality of life, and gut microbiota in patients with UC and IBS.

## 1. Introduction

Irritable bowel syndrome (IBS) and ulcerative colitis (UC) are gastrointestinal disorders with rising global prevalence, especially in industrialized and Western countries, where inflammatory bowel disease (IBD) affects more than 3% of the population [1]. Both are chronic conditions that significantly reduce quality of life and often impair productivity [2]. Although IBS is a functional digestive disorder and UC is a chronic inflammatory disease, they share several gastrointestinal symptoms, such as abdominal pain, bloating, flatulence, and changes in bowel habits [1,3]. It has been mentioned that IBS is a multifactorial disorder associated with genetic and epigenetic factors, alterations in the gut microbiota, low-grade inflammation, gastrointestinal motility disorders, and disruption of the brain–gut axis [4]. Similarly, UC is a disease of multifactorial etiology in which genetic susceptibility and environmental factors, such as diet, contribute to the development and maintenance of intestinal inflammation [5].

Persistent symptoms often lead patients to modify their diets in search of relief, since certain foods are known to trigger discomfort [6]. To help manage these symptoms, a diet low in fermentable oligosaccharides, disaccharides, monosaccharides, and polyols (FODMAPs) has been suggested. The low-FODMAP diet (LFD) is a therapeutic strategy for managing patients with gastrointestinal disorders [7]. This diet has been reported to affect other clinical outcomes. By reducing osmotic load and the availability of rapidly fermentable substrates, the diet decreases luminal water content and gas production, leading to improvements in conditions such as diarrhea, abdominal distension, abdominal pain, and consequently, an improved health-related quality of life. On the other hand, since many high-FODMAP foods also serve as sources of prebiotic fiber and micronutrients, the restrictive phase of the diet can contribute to vitamin and mineral deficiencies, a particularly relevant point for patients with IBS and UC, who are already vulnerable to malnutrition. This raises questions about the long-term safety of the diet [8]. This diet has been extensively studied in patients with IBS and has been shown to reduce gastrointestinal symptoms, especially in those with diarrhea. Research involving IBD patients has also increased [1,2,9].

LFD has been shown to improve gastrointestinal symptoms in UC patients by reducing abdominal pain and bloating and enhancing quality of life, without changing stool consistency [10]. The same benefits have been demonstrated in patients with IBD in remission. However, the evidence for these populations remains limited and inconsistent [11]. Although LFD has been reported to relieve gastrointestinal symptoms, concerns about the nutritional effects of long-term restriction of FODMAPs are increasing. This dietary pattern involves limiting the intake of micronutrients such as calcium, iron, folate, and vitamin D, especially by excluding dairy products, legumes, and whole grains. Additionally, reducing prebiotic fiber intake may negatively impact gut microbiota composition, including beneficial strains such as *Bifidobacterium* spp. [6,12].

Few studies have examined the nutritional effects of an LFD, particularly in Latin American populations. Therefore, the main goal of this study was to assess the impact of a 10-week LFD on nutritional status and quality of life in patients with IBS and UC. The study also explored how an LFD affects gut microbiota.

## 2. Materials and Methods

### 2.1. Trial Design

**Figure 1:**
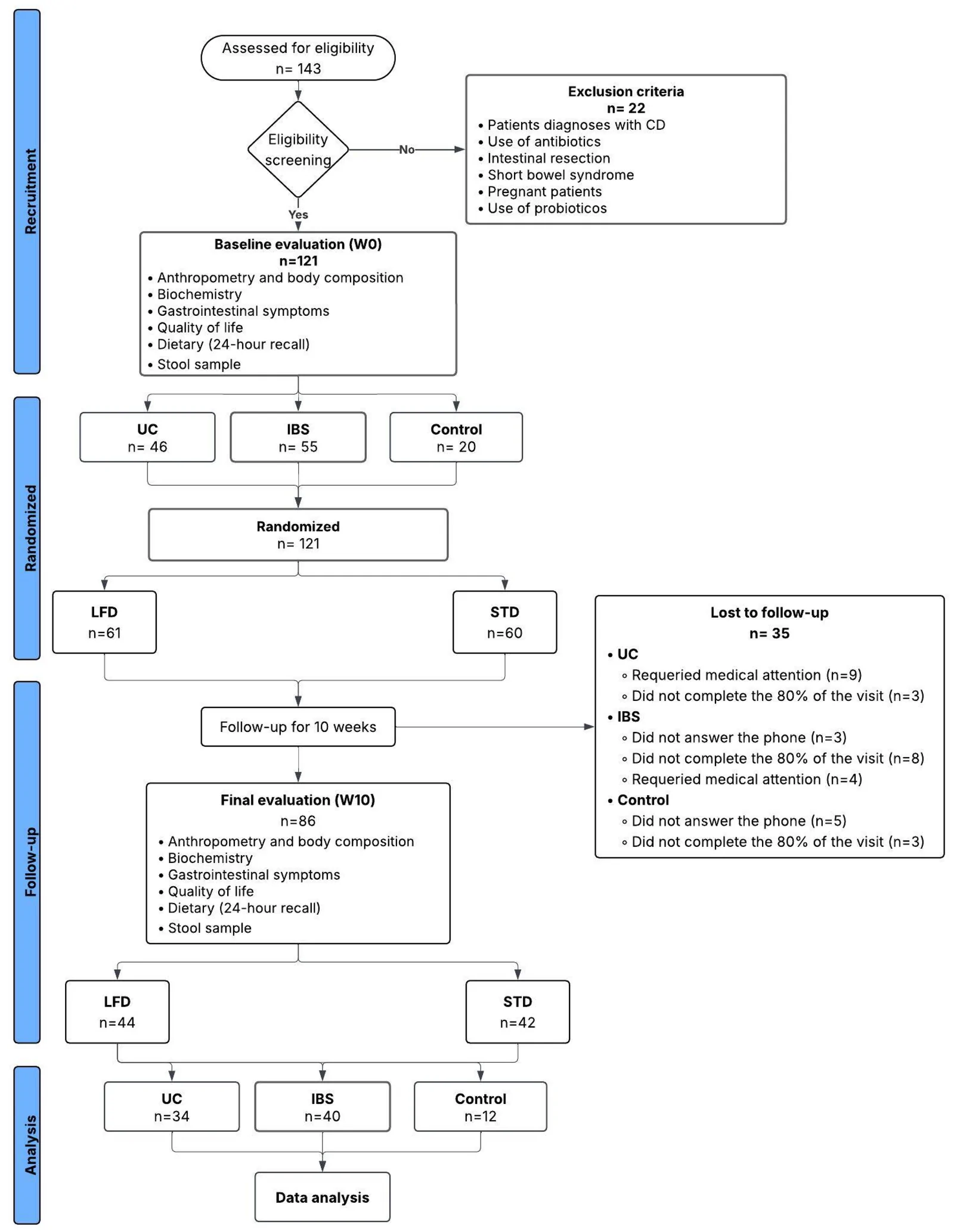
The flowchart shows the progress of participants who were recruited, randomized, and followed up during the 10-week study, according to the CONSORT (Standardized Standards for Reporting Clinical Trials) guidelines.

A randomized, controlled, one-blind, parallel-group study was conducted in accordance with the Consolidated Standards of Reporting Trials (CONSORT) guidelines [13], and the trial was registered at ClinicalTrials.gov (identifier: NCT04143633) (Figure 1).

### 2.2. Ethical Considerations

The ethics committee of the Hospital General de México “Dr. Eduardo Liceaga” (HGMEL) approved this study with the number DI/17/301/03/084. All participants received an explanation of the study’s purpose and procedures, and finally, everyone signed an informed consent form. According to Article 17 of the Regulations of the General Health Law on Health Research in Mexico, this study was classified as research involving more than minimal risk.

### 2.3. Eligibility Criteria for Participants

The inclusion criteria were that subjects should be Mexican men and women aged 18 to 59 years with a diagnosis of IBS according to the Rome III criteria. Because patient recruitment began before the publication and widespread adoption of the Rome IV criteria, the IBS subtype was not systematically recorded; therefore, participants could not be classified as IBS-C, IBS-D, IBS-M, or IBS-U for the present analysis. UC had been previously diagnosed by a hospital-based gastroenterologist and histologically confirmed. Baseline total or partial Mayo scores were not systematically collected as part of the study assessments and were therefore unavailable for reporting. Participants had a Body Mass Index (BMI) between 18.5 kg/m^2^ and 29.9 kg/m^2^. Individuals with a BMI ≥ 30 kg/m^2^ were excluded to reduce potential heterogeneity in gut microbiota composition associated with obesity and its related metabolic and inflammatory alterations. Each participant signed an informed consent form and agreed not to consume alcohol or probiotics during the study. Exclusion criteria included individuals with a history of anemia, malnutrition, short bowel syndrome, intestinal resection, Crohn’s disease, recent antibiotic use within two weeks before the study, regular use of probiotics or digestive enzymes, nutritional supplements, diagnoses of diabetes, hypertension, pregnancy, and lactation. The elimination criteria included participants who missed more than 80% of scheduled visits, those who used antibiotics, probiotics, or gastrointestinal enzymes during the 10-week period, and UC patients with disease complications requiring medication. Participants were recruited at the Laboratory of Proteomics and Metabolomics, Research Division, at the HGMEL (19°24′46.94″ N, 99°9′6.97″ W). The control group included healthy individuals without metabolic diseases or gastrointestinal symptoms.

### 2.4. Interventions

Their total energy expenditure was subsequently estimated using the Harris–Benedict formula. The diet was distributed as 55% carbohydrates, 20% proteins, and 25% lipids, with a weekly menu divided into five meals. Each volunteer was randomly assigned to two types of diet for ten weeks: LFD (less than 15 g of FODMAP per day) or Standard Diet (STD) (more than 20 g of FODMAP per day). Both diets were prescribed individually based on estimated energy requirements and had similar macronutrient distributions. Diets restricted the consumption of legumes, full-fat dairy products, cruciferous vegetables, and condiments, and participants were asked to limit themselves to foods included in the eating plan. The patients did not know each other, so none of them knew what type of diet they had been assigned.

All volunteers were evaluated at the beginning of the study, week 0 (W0), and after 10 weeks (W10) on the diets.

### 2.5. Outcomes

A dedicated nutritionist took body measurements, performed anthropometric assessments, and administered the questionnaire.

The primary outcomes were changes in nutritional status after 10 weeks of intervention. Nutritional status was assessed through anthropometric measurements, body composition, and biochemical markers. The secondary outcomes included changes in bowel habits, gut microbiota composition, and quality of life.

### 2.6. Biochemical Outcomes

For biochemical assessment, patients fasted for 8 to 10 h. Blood samples were collected to measure serum cholesterol, LDL-c, HDL-c, triglycerides, albumin, glucose, insulin, vitamin B12, calcium, iron, urea, creatinine, and complete blood counts. In UC patients, the erythrocyte sedimentation rate (ESR) and C-reactive protein (CRP) were also measured. These samples were collected and analyzed by personnel from the HGMEL central laboratory. Samples were taken at W0 and W10 of the study.

### 2.7. Anthropometric Outcomes

Assessment was conducted with the patient lying supine on a bed, wearing light clothing, and without metal objects in the hands, arms, or legs, using RJL Quantum III System equipment (RJL Systems Inc., Clinton Township, MI, USA). Body composition was measured at W0 and W10; resistance and reactance were used to calculate the phase angle (PhA) and estimate fat mass (FM), skeletal muscle mass (SMM), total body water (TBW), extracellular water (ECW), and intracellular water (ICW). We used the formula for the Hispanic population [14]. Additionally, anthropometric measurements, including weight, height, and circumferences of the waist, hips, arms, and chest, were obtained using a standardized anthropometric tape.

### 2.8. Clinic and Dietetic Outcomes

Clinical and dietary outcomes were evaluated at W0 and W10. Dietary adherence was measured using 24 h dietary reminders collected during follow-up visits. To assess stool consistency and bowel habits, a gastroenterologist and a nutritionist used a Bristol Stool Scale questionnaire [15]. The presence of constipation was defined as the report of stools corresponding to Bristol types 1–2, whereas the presence of diarrhea was defined as the report of stools corresponding to types 5–7 during the assessment period. These variables were recorded independently and were not mutually exclusive because participants could report both hard and loose stools, reflecting alternating or mixed bowel habits.

Additionally, participants’ quality of life was evaluated using the World Health Organization Quality of Life Questionnaire (WHOQOL-BREF). This validated tool measures physical health, psychological well-being, social relationships, and environment [16].

The IBS Severity Scoring System was not used because the original study protocol did not include multidimensional symptom severity as an outcome. The clinical assessment was limited to stool consistency and bowel habits, evaluated with the Bristol Stool Form Scale.

### 2.9. Gut Microbiota Outcomes

Stool samples were collected from UC and IBS participants at W0 and W10. Samples were placed in sterile stool containers and immediately frozen upon receipt until analysis. DNA was extracted from 150 mg of stool using the DNA PowerFecal DNA kit (Qiagen, Hilden, Germany) according to the manufacturer’s instructions. The quality and quantity of the extracted DNA were assessed with a spectrophotometer (DS-11FX, DeNovix, Wilmington, DE, USA). For high-throughput sequencing, a 16S rDNA library was prepared. A PCR reaction with a total volume of 20 μL was performed for each sample to amplify the V3 hypervariable region of the 16S rRNA gene, with a different barcode added to each sample, as previously reported [17]. The reaction buffer contained 200 µM dNTPs, 0.5 µM of each primer, 2 mM MgCl2, 0.02 U/μL PhusionTM high-fidelity DNA polymerase (ThermoScientific, Waltham, MA, USA), 1X Phusion HF Buffer, and 10 ng of DNA. All PCR reactions were conducted on a CFX96 thermal cycler (Bio-Rad, Hercules, CA, USA) with an initial denaturation at 98 °C for 3 min, followed by 30 cycles (98 °C for 12 s, 62 °C for 15 s, and 72 °C for 10 s), and a final extension at 72 °C for 5 min. The expected amplicon (~281 bp) was quantified by densitometry using 2% agarose gels, and equal amounts of each barcode were combined to generate the library.

### 2.10. High-Throughput DNA Sequencing

The library was purified using 2% agarose gel stained with SYBR GOLD DNA (E-Gel™ EX, 2%, Invitrogen™, Cat. G401012, Waltham, MA, USA). The final concentration and fragment sizes were assessed with the 2100 Bioanalyzer Instrument (Agilent Technologies, Santa Clara, CA, USA). Emulsion PCR was performed using the Ion PGM HI Q view OT2 Kit (Cat. A29900, Life Technologies, Carlsbad, CA, USA). Sequencing was carried out with the Ion PGM HQ view SEQ kit (Cat. A30044, Life Technologies, Carlsbad, CA, USA), the Ion 318 Kit V2 Chip (Cat. 4488146, Life Technologies, Carlsbad, CA, USA), and the Ion Torrent PGM system v4.0.2. After sequencing, the PGM software filtered the reads to exclude polyclonal sequences (homopolymers > 6) and low-quality reads (quality score ≤ 20).

Sequencing was performed in a single run, with no apparent batch effects. Negative controls did not yield relevant reads and were excluded from subsequent analyses. Additionally, a positive control with probiotics containing *Bifidobacterium* and *Lactobacillus* was used, demonstrating the expected taxa without abnormalities.

### 2.11. Microbiota Data Processing and Analysis

Amplicon sequence variants (ASVs) were identified from quality-filtered sequencing reads using the QIIME2-2024.2 pipeline with the DADA2 plugin (–p-trunc-len: 172 nucleotides). A summary of demultiplexed read counts prior to DADA2 processing and the read frequency histogram are provided in Table S2 and Figure S1, respectively. A truncation length of 172 nucleotides was chosen based on the sequencing quality profile, as this length retained the most informative features and preserved similar read lengths across samples, which are provided in Figure S2 and Table S3, respectively.

Representative sequences were taxonomically annotated using BLAST alignment in QIIME2 against the Greengenes2 database version 2022.10, with a 97% identity threshold for taxonomic assignment. No machine learning taxonomic classifier was employed, so no confidence threshold was established. Assignments were maintained at the genus level in subsequent analyses, considering the resolution of the V3 16S rRNA amplicon [18]. Processed microbiota data were imported into phyloseq 1.52.0 [19] objects using the qiime2R package (v0.99.6). Sample metadata were organized to define experimental groups (IBS, UC, Control), dietary interventions (LFD and STD), timepoints (W0 baseline, W10), and subject identifiers.

The observed number of species, alpha diversity indices (Shannon, Simpson, Fisher), and correlations between genus-level abundances and clinical metadata (Spearman correlation; Benjamini–Hochberg-adjusted *p*-value < 0.05) were calculated. Differential abundance patterns were visualized using violin plots with individual data points, and log-fold change estimates were shown with error bars. All analyses were conducted in R 4.5.1 (13 June 2025) using the packages phyloseq 1.52.0, microbiome 1.30.0, tidyverse 2.0.0, and ggpubr 0.6.1.

Beta diversity was also assessed using Aitchison distance and visualized through ordering plots. A separate PERMANOVA analysis was performed for the UC and IBS cohorts to assess differences in microbial community composition by time and diet, using the distance~time + diet model.

Low-prevalence and low-abundance taxa were filtered before analysis, keeping only taxa present in at least 90% of samples with a mean relative abundance greater than 0.01%. Samples with fewer than 100 reads were excluded. ASV count summaries before and after filtering are provided at the sample and taxon levels in Tables S4 and S5, respectively.

The core microbiota—taxa found in at least 90% of samples with an average relative abundance of at least 1%—were identified for each cohort and visualized using CLR-transformed, hierarchically clustered heatmaps (Ward’s method). Differential abundance testing was performed separately for UC and IBS cohorts using ANCOMBC2 2.10.1 [20]. A method specific to compositional microbiome data was used, incorporating bias correction and variance regularization. The ANCOM-BC2 model included variables such as time, diet, and their interaction (time × diet) to assess whether variations in taxon abundance over time depended on the dietary intervention. Analyses were performed separately for each disease cohort, where sample size allowed, considering a significance threshold of q < 0.05.

### 2.12. Sample Size

The sample size was calculated using GPower v. 3.1.9.2, with a two-tailed test for independent means, assuming an effect size of 0.5, an alpha level of 0.05, and 80% power, resulting in a total of 64 participants per intervention arm. The effect size was based on previous findings by Stadaucher et al. [21], who studied the clinical effects of an LFD in functional gastrointestinal disorders.

The sample size was initially determined considering the main clinical/nutritional outcome of the dietary intervention and was not specifically designed to analyze disease subgroups, repeated measures interactions, microbiota outcomes, biochemical variables, stool consistency and bowel habits, or aspects of quality of life. Therefore, subgroup analyses, microbiological findings, and various secondary outcomes should be interpreted as exploratory.

### 2.13. Randomization

Participants were randomly assigned within each diagnostic cohort (IBS, UC, and healthy controls) to receive either the LFD or the STD in a 1:1 allocation ratio. The randomization sequence was generated by the principal investigator using a computer-generated randomization schedule created with Randomization.com (http://www.randomization.com). Fixed permuted blocks of four participants were used within each diagnostic stratum to ensure balanced allocation between the intervention groups.

The registered dietitian was responsible for delivering the individualized dietary intervention according to the assigned treatment group and therefore had access to the randomization list.

### 2.14. Blinding

The study was conducted as a single-blind trial, with participant masking. Participants received personalized dietary plans but were not informed whether they were on the LFD or STD, nor were they explicitly informed of the FODMAP content or diet classification during the process. Due to the nature of the dietary intervention, allocation concealment from the dietitian was not feasible, and no independent allocation-concealment mechanism was implemented.

Outcome assessors, laboratory personnel, and investigators responsible for statistical analyses remained blind to group allocation throughout the study.

### 2.15. Statistical Analysis

Statistical analysis was carried out on the per-protocol population, defined as participants who completed the 10-week intervention and did not meet any elimination criteria or have major protocol deviations. SPSS v25 software was used for analysis. To assess the normality of the quantitative data, a Kolmogorov–Smirnov test was conducted. Descriptive statistics involved measures of central tendency and dispersion. For inferential statistics, Chi-square tests, related *t*-tests, and independent *t*-tests were used.

Between-group comparisons of continuous anthropometric and body-composition outcomes at W10 were performed using analysis of covariance (ANCOVA), with dietary intervention as the fixed factor and the corresponding baseline value as the covariate. Adjusted means, between-group differences, 95% confidence intervals, and *p*-values were reported, and the assumptions of the ANCOVA models were evaluated.

To compare variables between W0 and W10, the Wilcoxon signed-rank test was applied for quality-of-life domains, and changes in stool consistency and bowel habits were analyzed with the chi-square test. In all tests, *p* < 0.05 was considered statistically significant.

## 3. Results

### 3.1. Participant Flow

In total, 143 patients were assessed for eligibility, of whom 22 met the exclusion criteria. Of the 121 eligible participants, 61 were randomly assigned to the LFD and 60 to the STD. During the 10-week follow-up, 35 participants discontinued the study, resulting in a final per-protocol sample of 86 participants, as shown in Figure 1.

### 3.2. Baseline Results

A total of 86 patients completed the ten-week intervention and were included in the per-protocol analysis: 40 with IBS, 34 with UC, and 12 healthy controls. Within the IBS group, 23 were assigned to LFD and 17 to STD; within the UC group, 15 were assigned to LFD and 19 to STD; and within the healthy control group, 6 were assigned to each dietary intervention.

Baseline characteristics by dietary randomization within each diagnostic group are presented in Table 1. The IBS group was predominantly female across both intervention groups. Mean age was similar between the LFD and STD groups in participants with IBS (40 ± 11 vs. 41 ± 10 years) and UC (37 ± 11 vs. 37 ± 11 years), whereas the corresponding values in healthy controls were 31 ± 10 and 28 ± 10 years, respectively. Baseline BMI was also similar between dietary groups in the IBS and UC cohorts. Some numerical differences in body composition and certain biochemical parameters were observed, particularly among healthy controls; however, these estimates should be interpreted with caution, as this subgroup included only six participants per intervention arm.

### 3.3. Anthropometric, Body Composition, Hematological, and Biochemical Parameters

Anthropometric, body composition, hematological, and biochemical values recorded at baseline and at week 10 are detailed in Supplementary Table S6. Differences between W0 and after LFD and STD (W10) are shown, separated by group. In the IBS group, a statistically significant rise in urea was observed following LFD, while in the STD group, BMI increased (W0 = 26 ± 3.8 kg/m^2^ vs. W10 = 26.4 ± 0.9 kg/m^2^, *p* = 0.023). In the UC group, changes were observed only in patients on the LFD, with a significant decrease in hip measurement (W0 = 99.1 ± 8.6 cm vs. W10 = 97.5 ± 8.3 cm, *p* = 0.040) and an increase in PhA (W0 = 6.2 ± 0.6 grades vs. W10 = 6.7 ± 1.1 grades, *p* = 0.026). In the control group, differences were observed among patients on LFD, with an increase in eosinophils (1.3 ± 0.8% vs. 1.5 ± 0.9%, *p* = 0.046).

Comparisons between groups, adjusted for baseline anthropometric and body composition results, are presented in Table 2. After adjusting for baseline values, no statistically significant differences were observed between the LFD and STD groups in the IBS, UC, or healthy control cohorts. In IBS, the weight- and BMI-adjusted differences were close to, but did not reach, the threshold for statistical significance. Similarly, in UC, the differences in hip circumference and fat mass did not reach statistical significance. In the UC cohort, a significant interaction was observed between baseline PhA and diet, suggesting that the difference between interventions depended on the initial PhA value.

### 3.4. Stool Consistency and Bowel Habits and Quality of Life

We also observed differences in quality of life and changes in bowel movement consistency with LFD (Table 3). In the IBS group, significant improvements occurred after 10 weeks (W0 vs. W10) in patients who followed LFD in quality of life (3 ± 1 vs. 4 ± 1, *p* = 0.003) and overall health perception (2 ± 1 vs. 3 ± 1, *p* = 0.016), physical health (12.8 ± 2.4 vs. 14.6 ± 2.5, *p* = 0.005), psychological health (13.1 ± 3.2 vs. 14.7 ± 2.4, *p* = 0.007), social relationships (11.8 ± 4.4 vs. 13.6 ± 3, *p* = 0.034), and environment (11.8 ± 1.9 vs. 12.8 ± 2.4, *p* = 0.028). Meanwhile, IBS patients on the STD showed increases in overall health perception (2 ± 1 vs. 3 ± 1, *p* = 0.029) and physical health (12.9 ± 2 vs. 14.1 ± 2.8, *p* = 0.001). Among UC patients who received STD, improvements were observed in quality of life (3 ± 1 vs. 4 ± 1, *p* = 0.029) and overall health perception (3 ± 1 vs. 4 ± 1, *p* = 0.039). The control group, after LFD, showed changes in patients’ environmental conditions (17.1 ± 1.9 vs. 19.2 ± 0.7, *p* = 0.046).

### 3.5. Microbiota Outcomes

**Figure 2:**
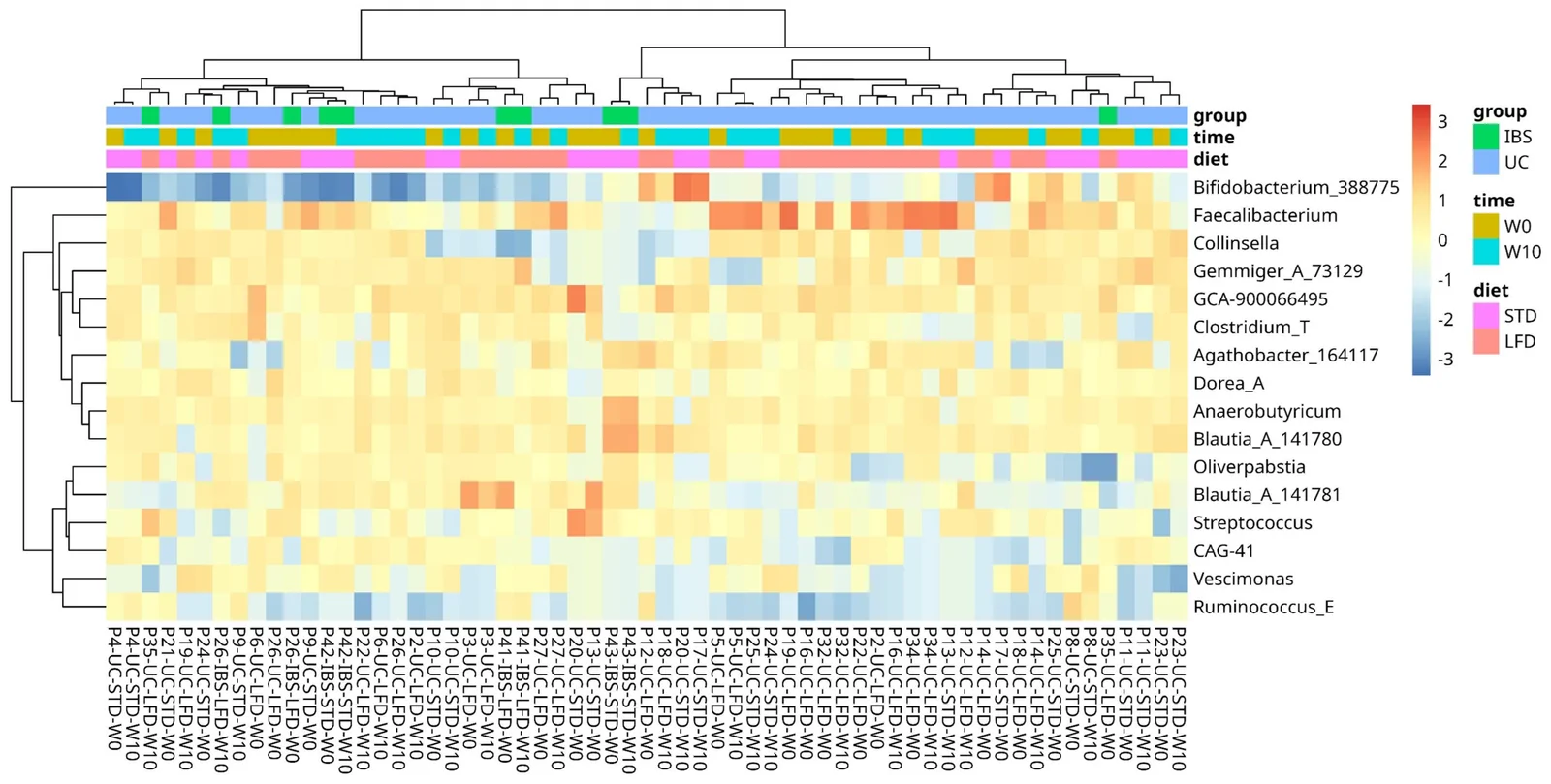
Core microbiota heatmap of 90% genus prevalence and 1% abundance. Samples are shown in the columns, while the genus is in the rows. Additional top-color labels are based on sample metadata by group (IBS, UC), time (W0, W10), and diet (STD, LFD).

**Figure 3:**
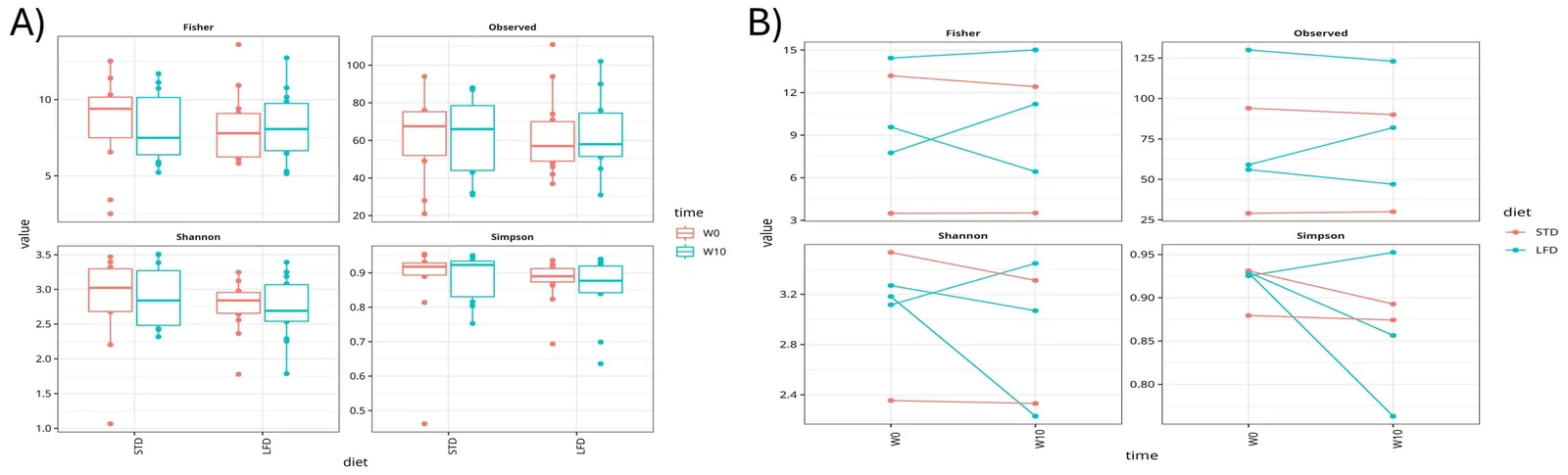
(**A**) Alpha diversity boxplot for individuals with UC. The *y*-axis shows the alpha-diversity values for the Fisher, Observed, Shannon, and Simpson indices. (**B**) Alpha diversity dotplot for the individuals with IBS. The *y*-axis shows the alpha-diversity values for the Fisher, Observed, Shannon, and Simpson indices. Connecting dots in the graph corresponds to the same individual evaluated at different times. The *X*-axis shows the time of the intervention (W0, W10), and the color maps the dietary intervention (STD, LFD).

We performed an exploratory analysis to assess bacterial diversity, using fecal samples from UC and IBS patients exposed to LFD versus STD. For UC, we included 23 samples from STD (12 at W0 and 11 at W10) and 28 samples from LFD (14 at W0 and 14 at W10). For IBS, we included 3 samples from STD (2 at W0, 2 at W10, and 1 on both) and 6 samples from LFD (3 at W0 and W10). Figure 2 displays the core bacterial genera found in at least 90% of the samples with ≤1% abundance. The samples are randomly distributed across the entire dataset, with no variable associated with clustering patterns. Similarly, we examined the impact of the interventions on microbial diversity in W10 samples from UC and IBS patients. No significant differences in alpha diversity indices were observed in the UC group across diets (Figure 3). For the IBS group, due to the small sample size, we could not perform the same detailed analysis and instead examined trends in changes from W0 to W10 for both diets. Still, it remains unclear whether diversity increased or decreased after the intervention. Additionally, we investigated whether patients with IBS and UC exhibit characteristic gut microbiota profiles, but no clustering was observed by pathology, as shown in Figure S3 (Supplementary Material).

In the Figure S4 includes beta-diversity analysis using Aitchison distance. PERMANOVA results indicated no significant differences related to time or diet in either UC samples (Pr(>F) = 0.65, F = 0.92, R^2^ = 0.037) or IBS samples (Pr(>F) = 0.52, F = 0.99, R^2^ = 0.22).

**Figure 4:**
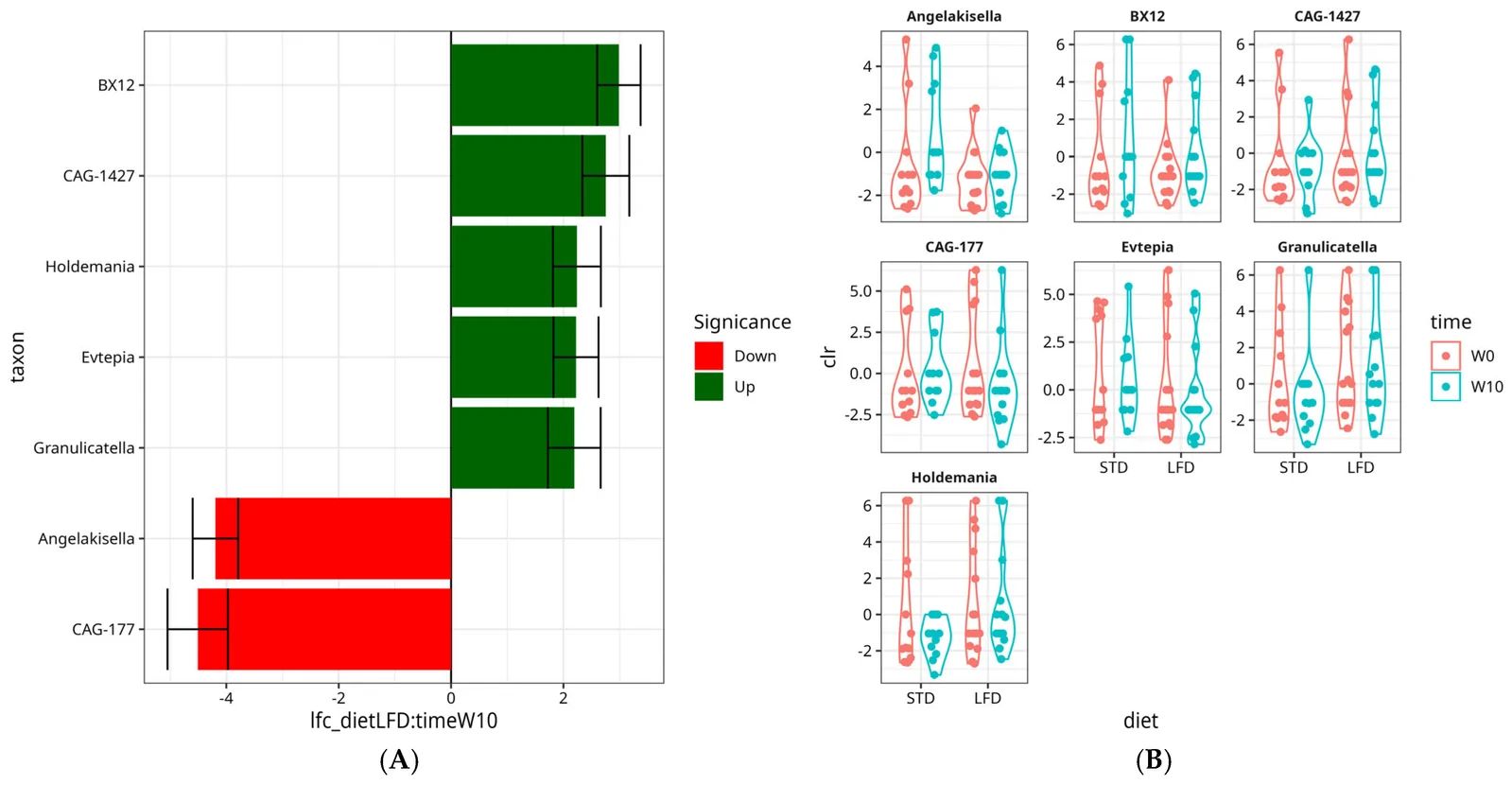
(**A**) Barplot showing LFC of differentially abundant bacteria. The effect of the LFD at W10 (lfc_dietLFD: timeW10) was tested using the model time × diet, using ANCOM-BC, and only genera with q-values < 0.05 were included in the graph. (**B**) Violin plot showing the distribution of the same significant features in the discrete groups; *X*-axis shows dietary intervention (STD, LFD). Color indicates time (W0, W10). Error bars in the barplot represent standard errors. Detailed effect sizes, and adjusted *p*-values for main effects and the interaction term are provided in Table S1.

To address the central question of whether specific bacteria changed after ten weeks on the LFD, and which bacteria were involved, both ALDEx2 and ANCOM-BC tests were applied. These tests were only feasible in the UC group, which had an adequate sample size. Although ALDEx2 did not yield significant results, ANCOM-BC provided fascinating insights into specific bacterial genera that changed during the intervention in the UC group. This is detailed in Figure 4, where bacteria from the genera BX12, CAG-1427, Holdemania, Evtepia, and Granulicatella were overly abundant after W10 of the LFD intervention, while Angelakisella and CAG-177 showed lower abundance. Among these, Holdemania, Granulicatella, and CAG-1427 decreased in abundance from W0 to W10 on an STD (only CAG-1427 showed a biologically relevant effect; see Table S1), while Angelakisella increased. Additionally, it is worth noting that the abundance of CAG-177 (decreased), *Angelakisella* (increased), CAG-1427 (decreased), and BX12 (decreased) differed between the STD and LFD groups at W0 (Table S1).

### 3.6. Harms

During follow-up, no adverse events directly attributable to any intervention were documented. Thirteen participants dropped out of the study because they required medical attention during follow-up. The most common reasons were episodes of gastroenteritis requiring antibiotic treatment and worsening of gastrointestinal symptoms or disease activity. However, given the underlying gastrointestinal conditions of the study population, it was not possible to determine whether these events were directly related to dietary intervention or reflected the natural course of the disease.

Additional losses occurred during follow-up because participants did not complete at least 80% of scheduled visits or could not be contacted. No participants dropped out of the study due to confirmed diet-related adverse effects.

## 4. Discussion

Gastrointestinal symptoms caused by FODMAPs result from their poor digestion in the intestine and fermentation by the colonic microbiota. Magnetic resonance imaging has been used to visualize the gastrointestinal tract and demonstrate that the amount of poorly digested FODMAPs increases water in the small intestines and, consequently, colonic gas in both healthy people and those with IBS. Visceral hypersensitivity is a key factor in the development of gastrointestinal symptoms [22].

### 4.1. Effects on Body Composition in IBS and UC

In this study, we assessed the effect of LFD on the nutritional status of patients with IBS and UC and its influence on gut microbiota composition. At baseline, we found significant differences in body composition among patients with IBS, UC, and healthy controls. Previous research reports that IBD patients have lower SMM and muscle strength compared to healthy controls [23], and that sarcopenia is more common in this group [24]. Observational studies suggest that sarcopenia may be present in up to 37% of UC patients [25], with disease extent linked to muscle mass loss [26]. Notably, reductions in SMM can occur even when BMI is normal, as seen in this study, where no patients were classified as “malnourished” by BMI. Similarly, IBS patients have also been shown to have lower muscle mass compared to healthy adults. Chapa et al. conducted a prospective, exploratory study involving 16 patients with IBS and 20 healthy controls; body composition was measured using bioelectrical impedance analysis. The results indicated that IBS patients had lower FFM (64.1% vs. 74.1%, *p* = 0.001), TBW (46.8% vs. 53.7%, *p* = 0.002), and PhA than controls, along with a borderline correlation between FFM and symptom severity, particularly in women. These findings align with ours, showing that IBS patients had reduced SMM, TBW, ICW, and ECW compared with healthy controls, despite no differences in BMI [27]. However, after adjustment for baseline values, no statistically significant differences in anthropometric or body-composition outcomes were observed between the LFD and STD groups.

PhA remains a clinically relevant indicator because it has been associated with nutritional status, cellular integrity, cell mass, and fluid distribution. Recent studies have reported correlations between PhA, FFM, and SMM [28]; in UC patients, those with better nutritional status also have higher PhA values [29]. A systematic review observed that PhA not only indicates SMM but also the quality and health of the cell membrane, as well as the fluid balance between the ICW and ECW compartments, which are essential elements for cellular integrity [30]. However, since inflammatory cytokines, muscle function, and other mechanistic markers were not assessed, the physiological pathways underlying these changes remain speculative and require further investigation.

Previous studies have indicated a connection between sarcopenia and the gut microbiota, especially the gut–muscle axis. In a narrative review, several mechanisms have been outlined by which an imbalanced gut microbiota might encourage muscle loss, including increased intestinal permeability and systemic inflammation, fewer short-chain fatty acid-producing bacteria, lower levels of IGF-1, mitochondrial dysfunction, and abnormalities in insulin sensitivity [31].

### 4.2. Effects on Diarrhea and Quality of Life

Our within-group analysis indicated a decrease in the proportion of participants experiencing diarrhea after 10 weeks of following an LFD. Although a numerical reduction was seen among participants with IBS, it did not reach statistical significance. These results should be interpreted with caution because diarrhea was assessed as a non-exclusive bowel habit variable, and within-group analyses do not confirm that changes exceed those seen with STD. Nonetheless, the reduction aligns with prior evidence suggesting a potential benefit of LFD for diarrhea. A 14-day randomized, double-blind, multicenter trial in hospitalized patients receiving enteral nutrition compared formulas with low, moderate, and high FODMAP levels. The study found that the LFD group experienced a significant decrease in diarrhea episodes compared to higher FODMAP groups. Early improvements in nutritional markers were also noted: prealbumin levels increased significantly, and BMI improved in both the low- and high-FODMAP groups, indicating that the reduction in diarrhea with LFD may support short-term nutritional recovery [32].

Regarding quality of life, exploratory within-group improvements in several WHOQOL-BREF domains were observed among participants with IBS receiving LFD. However, improvements were also observed with STD, and these analyses do not demonstrate that LFD was superior to STD. In UC, STD improved the overall quality of life and health perception. Several studies support the idea that LFD improves patients’ quality of life, as shown by Kortlever T. et al., who reported long-term enhancements in quality of life and gastrointestinal symptoms, along with reduced fatigue and anxiety/depression, and increased happiness and vitality after LFD [33].

It has been suggested that improved quality-of-life perceptions may be linked to reductions in digestive discomfort, a lower emotional burden, and an increased sense of control and autonomy. Learning to identify food triggers may reinforce patients’ sense of mastery over their condition and contribute to psychological and social well-being [22,34,35]. Nevertheless, the quality-of-life findings of the present study should be considered exploratory because they were based primarily on within-group comparisons.

### 4.3. Effects on Gut Microbiota Diversity

Regarding gut microbiota diversity, no significant changes in bacterial alpha-diversity indices were observed after 10 weeks of LFD among patients with UC. In the IBS group, the number of samples from which microbiota could be sequenced was much lower than in the UC group; nonetheless, we did not observe any trend in changes in alpha diversity between W0 and W10 with LFD. This aligns with previous studies where an LFD, while reducing gastrointestinal symptoms, did not always lead to broad changes in microbial diversity. Staudacher H. et al. reported that, in the short term (3–4 weeks of LFD), a decrease in Bifidobacteria spp. is observed, but in the long term (12 months), no difference from baseline is noted [36].

Our results show a decrease in Angelakisella after LFD in patients with UC. Although knowledge about this genus is limited, its abundance has been reported to correlate with intestinal serotonergic signaling (5-HT and its receptor 5-HT4R). In models of induced constipation, changes in Angelakisella were associated with alterations in serotonin production [37]. Other studies have found that Angelakisella is more abundant in animal models of obesity and markedly less abundant in obese mice fed a slowly digestible carbohydrate diet [38]. This suggests that the reduction in Angelakisella may be linked to changes in gastrointestinal physiology; however, the observed reduction should be interpreted with caution, and its physiological significance warrants further mechanistic investigation.

Serotonin is a key regulator of gastrointestinal motility and secretion, acting through multiple receptor subtypes, including 5-HT3 and 5-HT4 receptors. Emerging evidence suggests that gut microbiota and dietary factors may influence tryptophan metabolism and serotonergic signaling, thereby modulating gastrointestinal physiology. Although LFD has been shown to improve gastrointestinal symptoms, direct evidence linking these dietary interventions to specific changes in gut serotonin levels remains limited [39].

It is worth noting that we observed differences in some taxa in UC patients assigned to STD or LFD at week 0 (before the intervention). These differences could be due to chance or indicate the need for a larger sample size. Many taxa that showed differences in week 0 between the LFD and STD groups also showed differences after 10 weeks of LFD (CAG-177, CAG-1427, and Angelakisella). However, the presence of baseline differences limits the interpretation of the findings observed after the intervention. While several taxa that showed baseline differences (CAG-177, CAG-1427, and Angelakisella) also showed differences at week 10, the magnitude and direction of these changes were not entirely consistent across time points. Therefore, it is difficult to determine whether the differences observed at week 10 reflect the effects of the dietary intervention, pre-existing variability in microbiota composition, or a combination of both factors.

Among the taxa identified by ANCOM-BC, Holdemania has been associated with the production of short-chain fatty acids (SCFA), including acetate, propionate, and butyrate, metabolites involved in intestinal barrier integrity, immune regulation, and the maintenance of intestinal homeostasis [40].

ANCOM-BC, Holdemania and Granulicatella increased after 10 weeks of LFD treatment in patients with chronic UC. However, increased abundance of Holdemania has been associated with sodium dextran sulfate-induced colitis in experimental models. In a murine model of sodium dextran sulfate-induced colitis, Holdemania abundance increased in animals with colitis and decreased after treatment, supporting a possible association between this genus and intestinal inflammatory processes. These observations suggest that changes in Holdemania abundance may reflect changes in the microbial communities involved in intestinal inflammation [41]. Nevertheless, the specific biological role of Holdemania in UC remains incompletely understood and warrants further investigation. On the other hand, Granulicatella has been associated with adverse clinical outcomes in IBD. In patients with severe acute UC, a greater abundance of Granulicatella was observed in those who did not respond to exclusive enteral nutrition or corticosteroid therapy, and this abundance was associated with higher inflammatory markers, including CRP, as well as lower serum albumin levels. These findings suggest that Granulicatella may be related to a less inflammatory microbial profile and a poorer response to treatment [42].

We did not identify microbiota profiles specific to IBS or UC patients at baseline (W0), suggesting that both diseases share a standard gut microbiota profile, at least in the most abundant species. Although LFD has shown gastrointestinal benefits in conditions such as IBS and UC, reports indicate that 30–50% of patients are “non-responders” [40]. A challenge lies in identifying factors that can predict the response to LFD. It has been proposed that variability in the gut microbiome may be a key factor influencing the response to dietary treatment. The review by Chumpitazi et al., [43] describes baseline microbial signatures that differentiate responders from non-responders to the LFD, with saccharolytic bacteria enriched in responders in some cases. As explained by Valuer et al., [44] patients who responded to LFD showed enrichment for Bacteroides fragilis, Acinetobacter, Ruminiclostridium, Streptococcus, and Eubacterium. At the same time, non-responders exhibited enrichment of Clostridia, Negativicutes, Bacilli, Actinomycota, Anaerotruncus, Clostridiales, Shigella, and Escherichia.

Our findings, although exploratory, support the idea that patients who do not respond to LFD form a subgroup with unique pathophysiological and microbial features, emphasizing the need for more personalized dietary approaches tailored to the microbiome and its metabolic functions.

### 4.4. Strengths and Limitations

Our study is distinguished by integrating variables related to nutritional status, gut microbiota, and quality of life after LFD, as well as by a 10-week follow-up, which is rare in this type of intervention. Likewise, comparing responses between patients with IBS and those with UC provides valuable information on the differential response to this intervention in two populations that share gastrointestinal symptoms.

However, the main limitation is the small size of the control group, which limits comparison of the dietary response with that of a group without gastrointestinal symptoms. This may affect the generalizability of the results, so the findings should be interpreted with caution.

Another limitation concerns the analysis of the gut microbiota. The use of 16S ribosomal RNA (16S rRNA) sequencing offers limited taxonomic resolution and does not enable direct characterization of microbial functional pathways, restricting the biological interpretation of observed taxonomic changes [45]. Furthermore, microbiota samples were collected only at two points: week 0 and week 10. Due to the known temporal variability of the gut microbiota, more frequent longitudinal sampling, combined with metagenomic and metabolomic approaches, would provide a more comprehensive understanding of microbial dynamics and their relationship to clinical outcomes. The small sample size, particularly for the IBS group, represents an additional constraint.

The analysis was performed on the per-protocol population, including 86 out of 121 randomized participants. Data from the 35 participants who did not complete the week-10 assessment were excluded from the final database, preventing a reliable intention-to-treat analysis. As a result, potential attrition bias and the loss of randomization benefits cannot be ruled out, especially since some discontinuations were due to clinical worsening or medical needs. Additionally, baseline differences and the small size of disease-specific subgroups may have reduced the precision and influenced the interpretation of the treatment outcomes.

Body composition variables were estimated using BIA and should therefore be interpreted with caution. These estimates can be affected by hydration status, recent food and fluid intake, physical activity, bladder status, electrolyte balance, and gastrointestinal fluid losses. Although measurements were performed using the same equipment and a standardized body position, not all pre-measurement conditions were systematically recorded or controlled. Consequently, observed changes in estimated SMM may partly reflect changes in body fluid distribution.

This study assessed bowel habits with the Bristol Stool Scale, a straightforward and clinically useful measure of stool consistency. However, it doesn’t capture the full range of gastrointestinal symptoms, such as abdominal pain, bloating, urgency, or symptom severity. The IBS subtype was not consistently documented, and symptom severity was not measured using a validated multidimensional tool like the IBS Severity Scoring System.

Despite the use of an intention-to-treat approach, 35 of the 121 randomized participants did not complete the week-10 assessment. Because some losses were related to clinical deterioration or the need for medical treatment, residual attrition bias cannot be excluded. In addition, baseline imbalances and the limited size of the disease-specific subgroups may have reduced precision and affected the interpretation of the treatment effects.

Finally, multiple nutritional, clinical, and microbiota-related outcomes were evaluated throughout the study. Although these variables were predefined as part of the study objectives, no formal correction for multiple comparisons was applied. Therefore, findings with borderline statistical significance should be interpreted with caution, as the possibility of type I error cannot be completely excluded.

## 5. Conclusions

Our findings suggest that dietary intervention can improve gastrointestinal symptoms, quality of life, and certain nutritional parameters in patients with IBS and UC. These clinical changes occurred alongside modifications in the composition of the gut microbiota, leading us to infer that diet can influence multiple interconnected aspects of gastrointestinal health.

It is also important to recognize that some improvements were seen in participants who received STD. Therefore, part of the observed benefits may be attributable to the nonspecific effects of nutritional advice, increased attention to eating habits, or improved adherence to a structured eating plan, rather than to FODMAP restriction alone.

Growing evidence highlights the complex interactions between the gut microbiota, gastrointestinal function, nutritional status, and patient-reported outcomes, emphasizing that these aspects are closely interconnected in patients with IBS and UC.

The effects of LFD extend beyond symptom improvement and involve multiple domains, including quality of life, nutritional status/body composition, and gut microbiota composition.

These results support the idea that dietary intervention in patients with gastrointestinal symptoms is vital for their management, always with a personalized approach.

Figures summarize the multidimensional effects observed in this study, integrating changes in gastrointestinal symptoms, quality of life, body composition, and gut microbiota.

Studies with larger sample sizes, metabolomic profiling, and metagenomic analyses are needed to identify predictors of clinical response to LFD in UC and IBS.

## Figures and Tables

**Table 1:** Baseline characteristics by randomized dietary intervention within each diagnostic cohort.

Variable	IBS n = 40		UC n = 34		Healthy Controls n = 12	
	LFD n = 23	STD n = 17	LFD n = 15	STD n = 19	LFD n = 6	STD n = 6
**Demographic and anthropometric characteristics**						
Male, n (%)	1 (4.3)	0 (0)	6 (40)	6 (31.6)	2 (33.3)	3 (50)
Female, n (%)	22 (95.7)	17 (100)	9 (60)	13 (68.4)	4 (66.7)	3 (50)
Age, years	40 ± 11	41 ± 10	37 ± 11	37 ± 11	31 ± 10	28 ± 10
Weight, kg	66.2 ± 8.1	61.7 ± 8.8	61.6 ± 10.8	65.4 ± 12.3	70.4 ± 9.9	74 ± 12.3
BMI, kg/m^2^	26.9 ± 2.9	26.1 ± 3.5	24.3 ± 3.3	25.2 ± 3.5	24.7 ± 3.5	26.3 ± 2.1
Waist circumference, cm	84.5 ± 7.1	81.7 ± 8.3	79.9 ± 10.4	82.4 ± 10.7	80.9 ± 10.2	82.2 ± 8.1
Hip circumference, cm	101 ± 7.2	97.8 ± 7.6	98.6 ± 8.7	100.2 ± 8.7	103 ± 4.6	103.3 ± 5.9
Mid-upper arm circumference, cm	30.7 ± 2.8	30.2 ± 3.8	29.5 ± 3.1	31.2 ± 4.2	29.8 ± 2.2	32.1 ± 2.6
Chest circumference, cm	98 ± 5.3	97 ± 7.8	92.3 ± 6.8	96.4 ± 8.6	92.8 ± 7.1	97 ± 4.1
**Body composition**						
PhA, grades	6.2 ± 0.5	6.6 ± 0.8	6.1 ± 0.7	6.4 ± 0.9	6.4 ± 0.5	7.3 ± 0.9
TBW, L	27 ± 3	26.1 ± 2.9	31.4 ± 6.8	30.1 ± 8.7	33 ± 7.2	36.3 ± 8.1
ICW, L	13.9 ± 1.6	13.5 ± 1.4	17.2 ± 4.8	16.3 ± 5.5	17.7 ± 4.7	20.1 ± 5.4
ECW, L	13.1 ± 1.5	12.5 ± 1.5	14.2 ± 2.3	13.8 ± 3.3	15.2 ± 2.6	16.1 ± 2.7
SMM, kg	18.4 ± 3	17.9 ± 2.6	20.2 ± 4.7	20.5 ± 6.5	22.8 ± 4.4	26.9 ± 7.3
**Hematological parameters**						
Leukocytes, ×10^3^/uL	6 ± 2.4	6.3 ± 1.8	7.7 ± 2.8	7.1 ± 2.8	5.7 ± 1.4	7.7 ± 1.7
Lymphocytes, %	28.7 ± 8.3	30.5 ± 7.7	30.6 ± 10.1	28.8 ± 9.5	32.7 ± 5.3	30 ± 8.8
Eosinophils, %	2.5 ± 1.9	2.2 ± 1.4	1.04 ± 1	2.7 ± 2.4	1.3 ± 0.8	1.2 ± 0.5
Hemoglobin, g/dL	14.5 ± 1	14.4 ± 1.1	14.2 ± 2.1	14 ± 1.7	14.1 ± 1.6	16.1 ± 1.6
Hematocrit, %	43.3 ± 2.6	43.4 ± 3.2	43.9 ± 5.4	43.2 ± 4.3	42.7 ± 4.5	49.1 ± 4.9
MCV, fL	89.2 ± 4.9	91.4 ± 3.6	89.9 ± 9.5	87.6 ± 9.1	88.3 ± 5.3	93.5 ± 1.7
MCH, pg	29.8 ± 2	30.4 ± 1.2	29.1 ± 3.8	28.3 ± 3.6	29.2 ± 2.2	30.7 ± 0.6
Platelets, ×10^3^/uL, median (IQR)	251 (400)	295 (420)	317 (797)	288 (783)	302.5 (167)	253 (171)
Iron, µg/dL, median (IQR)	76 (90)	89 (209)	91.5 (118)	68 (139)	95.5 (131)	111.5 (148)
**Biochemical parameters**						
Glucose, mg/dL	94.5 ± 13.1	94.6 ± 10.7	98.7 ± 36.1	93.8 ± 6.5	101 ± 20.1	91.6 ± 6.1
Urea, mg/dL	25.4 ± 7.9	27.9 ± 6.4	27.2 ± 10.3	28.9 ± 6.2	26.8 ± 6.6	25.5 ± 6.3
Creatinine, mg/dL	0.6 ± 0.1	0.7 ± 0.1	0.8 ± 0.1	0.8 ± 0.1	0.8 ± 0.1	0.9 ± 0.1
Total Cholesterol, mg/dL	182.1 ± 30.9	191.4 ± 40.2	171.8 ± 35.5	177.2 ± 32.6	162.6 ± 16.1	163 ± 22.9
Triglycerides, mg/dL, median (IQR)	149 (346)	96 (225)	90 (613)	114 (161)	77 (185)	91.5 (154)
HDL Cholesterol, mg/dL	45.6 ± 9.2	54.7 ± 18.9	49.6 ± 15.7	50.1 ± 10.4	51.5 ± 5.5	47.5 ± 10.1
LDL Cholesterol, mg/dL	112.9 ± 24.8	120 ± 39	94.7 ± 16.6	111.1 ± 32	100.3 ± 8.5	105.6 ± 26.1
Albumin, g/dL	4.2 ± 0.2	4.2 ± 0.2	4.3 ± 0.4	4.4 ± 0.2	4.5 ± 0.2	4.6 ± 0.2
Calcium, mg/dL	9.4 ± 0.3	9.4 ± 0.3	9.5 ± 0.2	9.4 ± 0.2	9.7 ± 0.5	9.6 ± 0.3
Insulin, U/dL, median (IQR)	10 (13.6)	8.1 (16.3)	3.6 (8.1)	6.4 (27)	8.5 (31.8)	5.8 (11.9)
Vitamin B12, pg/dL, median (IQR)	436 (1394)	576 (1174)	383 (7903)	325 (2596)	306.5 (712)	338 (355)
ESR, mm/h, median (IQR)	—	—	8 (54)	7 (47)	—	—
CRP, mg/L, median (IQR)	—	—	3.6 (25)	4.1 (89.9)	—	—

IBS: Irritable Bowel Syndrome; UC: Ulcerative Colitis; BMI: Body Mass Index; PhA: Phase Angle; L: Liters; TBW: Total Body Water; ICW: Intracellular Water; ECW: Extracellular Water; SMM: Skeletal Muscle Mass; MCV: Mean Corpuscular Volume; MCH: Mean Corpuscular Hemoglobin; HDL: High-Density Lipoprotein; LDL: Low-Density Lipoprotein; ESR: Erythrocyte Sedimentation Rate; CRP: C-Reactive Protein. Continuous variables are presented as mean ± standard deviation or median (IQR), according to their distribution.

**Table 2:** Baseline-adjusted between-group comparisons of anthropometric and body-composition outcomes.

	LFD Adjusted Mean (95% CI)	STD Adjusted Mean (95% CI)	Adjusted Difference LFD–STD (95% CI)	*p*-Value
**IBS**				
Weight, kg	64.01 (63.13–64.89)	65.34 (64.31–66.37)	−1.33 (−2.71–0.05)	0.059
BMI, kg/m^2^	26.48 (26.11–26.86)	26.99 (26.55–27.42)	−0.51 (−1.08–0.07)	0.083
Waist circumference, cm	82.44 (81.16–83.73)	83.33 (81.83–84.83)	−0.89 (−2.87–1.10)	0.373
Hip circumference, cm ^†^	98.96 (97.91–100.02)	99.64 (98.40–100.87)	−0.67 (−2.32–0.97)	0.412
Mid-upper arm circumference, cm	30.68 (30.13–31.23)	30.20 (29.56–30.84)	0.48 (−0.37–1.32)	0.258
Chest circumference, cm ^†^	97.48 (96.56–98.39)	98.31 (97.25–99.38)	−0.83 (−2.24–0.57)	0.238
PhA, degrees	6.46 (6.22–6.70)	6.58 (6.30–6.86)	−0.12 (−0.49–0.26)	0.526
Fat mass, kg	25.43 (24.29–26.56)	25.86 (24.53–27.18)	−0.43 (−2.20–1.34)	0.625
TBW, L	26.97 (26.19–27.75)	27.82 (26.90–28.73)	−0.84 (−2.06–0.37)	0.166
ICW, L	13.99 (13.51–14.48)	14.40 (13.83–14.97)	−0.41 (−1.16–0.34)	0.278
ECW, L	12.98 (12.66–13.30)	13.41 (13.04–13.79)	−0.43 (−0.93–0.06)	0.084
SMM, kg	18.45 (17.62–19.29)	18.74 (17.76–19.71)	−0.28 (−1.57–1.00)	0.657
**UC**				
Weight, kg	63.99 (62.83–65.14)	64.95 (63.96–65.94)	−0.96 (−2.49–0.56)	0.207
BMI, kg/m^2^	24.62 (24.14–25.09)	25.01 (24.61–25.40)	−0.39 (−1.01–0.23)	0.210
Waist circumference, cm	80.82 (79.45–82.18)	82.05 (80.88–83.22)	−1.24 (−3.03–0.56)	0.170
Hip circumference, cm	98.15 (96.83–99.47)	99.83 (98.70–100.96)	−1.68 (−3.42–0.06)	0.057
Mid-upper arm circumference, cm	30.12 (29.35–30.89)	30.57 (29.91–31.23)	−0.45 (−1.48–0.57)	0.371
Chest circumference, cm	94.03 (92.70–95.37)	94.70 (93.56–95.84)	−0.67 (−2.44–1.11)	0.450
Fat mass, kg	19.55 (17.63–21.48)	21.93 (20.34–23.51)	−2.37 (−4.89–0.14)	0.064
TBW, L	31.42 (29.79–33.04)	30.95 (29.60–32.29)	0.47 (−1.64–2.58)	0.653
ICW, L	17.36 (16.34–18.37)	16.87 (16.03–17.71)	0.48 (−0.84–1.81)	0.459
ECW, L	14.05 (13.42–14.68)	14.08 (13.56–14.60)	−0.04 (−0.85–0.78)	0.930
SMM, kg	21.92 (20.46–23.37)	20.53 (19.32–21.73)	1.39 (−0.50–3.28)	0.144
**Healthy controls**				
Weight, kg	72.81 (71.48–74.15)	73.25 (71.92–74.59)	−0.44 (−2.34–1.46)	0.614
BMI, kg/m^2^	25.72 (25.23–26.21)	25.88 (25.39–26.37)	−0.16 (−0.87–0.55)	0.618
Waist circumference, cm	80.90 (78.11–83.69)	81.01 (78.22–83.81)	−0.11 (−4.06–3.84)	0.951
Hip circumference, cm	103.49 (101.58–105.40)	103.91 (102.00–105.82)	−0.43 (−3.13–2.27)	0.729
Mid-upper arm circumference, cm	30.32 (29.34–31.30)	30.73 (29.75–31.71)	−0.41 (−1.87–1.05)	0.543
Chest circumference, cm	94.40 (93.08–95.71)	95.64 (94.32–96.95)	−1.24 (−3.17–0.69)	0.180
PhA, degrees	6.92 (6.50–7.34)	6.90 (6.47–7.32)	0.03 (−0.62–0.67)	0.932
Fat mass, kg	23.13 (19.87–26.39)	21.82 (18.56–25.08)	1.31 (−3.32–5.94)	0.538
TBW, L	33.59 (29.91–37.26)	35.78 (32.11–39.45)	−2.19 (−7.46–3.07)	0.370
ICW, L	18.29 (16.05–20.53)	19.52 (17.29–21.76)	−1.23 (−4.45–1.98)	0.408
ECW, L	15.27 (13.85–16.69)	16.23 (14.81–17.65)	−0.96 (−2.99–1.07)	0.313
SMM, kg	24.55 (22.19–26.91)	25.48 (23.12–27.84)	−0.93 (−4.38–2.51)	0.556

Adjusted means and between-group differences were estimated with ANCOVA using the week-10 value as the dependent variable, dietary intervention as the fixed factor, and the corresponding baseline value as a covariate. Differences are expressed as LFD minus STD. A negative value indicates a lower adjusted week-10 value in LFD. ^†^ Levene’s test indicated heterogeneity of variance. LFD, low-FODMAP diet; STD, standard diet; IBS, irritable bowel syndrome; UC, ulcerative colitis; CI, confidence interval; PhA, Phase Angle; L, liter; TBW, Total Body Water; ICW, Intracellular Water; ECW, Extracellular Water; SMM, Skeletal Muscle Mass.

**Table 3:** Stool consistency, bowel habits, and Quality of Life by Group.

	IBS n = 40						UC n = 34						Control n = 12					
	LFD n = 23			STD n = 17			LFD n = 15			STD n = 19			LFD n = 6			STD n = 6		
Variable	W0	W10	*p*	W0	W10	*p*	W0	W10	*p*	W0	W10	*p*	W0	W10	*p*	W0	W10	*p*
Presence of diarrhea, n (%)	10 (43.5)	7 (30.4)	0.271	11 (64.7)	6 (35.3)	0.085	8 (53.3)	3 (20)	0.058	11 (57.9)	8 (42.1)	0.259	1 (16.7)	0 (0)	0.500	1 (16.7)	0(0)	0.500
Presence of constipation, n (%)	14 (60.9)	14 (60.9)	0.618	11 (64.7)	8 (47.1)	0.245	7 (46.7)	7 (46.7)	0.064	6 (31.6)	5 (26.3)	0.500	0 (0)	1 (16.7)	0.500	1 (16.7)	1 (16.7)	0.773
Quality of life	3 ± 1	4 ± 1	0.003	3 ± 0.1	3 ± 1	0.276	3 ± 1	3 ± 1	0.059	3 ± 1	4 ± 1	0.029	4 ± 1	5 ± 0.5	0.157	4 ± 1	5 ± 1	0.317
Overall perception of health	2 ± 1	3 ± 1	0.016	2 ± 1	3 ± 1	0.029	2 ± 1	2 ± 1	0.096	3 ± 1	4 ± 1	0.039	4 ± 1	4 ± 1	1.000	4 ± 1	4 ± 1	0.157
Physical health	12.8 ± 2.4	14.6 ± 2.5	0.005	12.9 ± 2	14.1 ± 2.8	0.001	12.6 ± 2.9	12.3 ± 2.6	0.472	13.4 ± 3.6	13.9 ± 3.1	0.248	18 ± 1.7	18.2 ± 1	0.655	16.72.9	16.7 ± 1.9	1.000
Psychological health	13.1 ± 3.2	14.7 ± 2.4	0.007	13.8 ± 2.2	14.3 ± 2.8	0.344	13.1 ± 2.8	13.8 ± 2.9	0.194	14.5 ± 3.3	15 ± 2.5	0.210	17.4 ± 1.2	18.4 ± 1.1	0.080	16.1 ± 2.4	16.2 ± 2.2	0.783
Social relationships	11.8 ± 4.4	13.6 ± 3	0.034	13.1 ± 3.7	13.5 ± 3.7	0.750	12.9 ± 3.6	13.8 ± 3.7	0.100	13.2 ± 3.6	13.9 ± 3.2	0.204	19.1 ± 1.1	19.7 ± 0.5	0.180	16.4 ± 3.2	17.1 ± 1.9	0.425
Environment	11.8 ± 1.9	12.8 ± 2.4	0.028	12 ± 2.1	12.8 ± 2.9	0.066	12.3 ± 2.5	13 ± 2.9	0.344	13.4 ± 2.7	13.6 ± 2.6	0.794	17.1 ± 1.9	19.2 ± 0.7	0.046	15.8 ± 1.8	16.5 ± 1.4	0.416

Diarrhea and constipation were recorded as independent, non-mutually exclusive variables. Participants reporting both Bristol types 1–2 and types 5–7 during the assessment period were included in both categories; therefore, percentages may sum to more than 100%. Percentages were calculated using the total number of participants in each diagnostic and dietary subgroup as the denominator. W0: baseline assessment at week 0; W10: Final evaluation at week 10; IBS: Irritable Bowel Syndrome; UC: Ulcerative Colitis; LFD: low FODMAP diet; STD: standard diet. For quality of life, overall perception of health, psychological health, social relationships, and environment, we use WHOQOL-BREF. For the analysis, we use the Wilcoxon test, and for stool consistency and bowel habits, we use the X2 test. Significant *p*-value < 0.05.

## Data Availability

The data presented in this study are openly available in Zenodo at 10.5281/zenodo.00000000 and https://www.ncbi.nlm.nih.gov/sra/PRJNA0000000 (accessed on 18 May 2026).

## Supplementary Materials

The following supporting information can be downloaded at: https://www.mdpi.com/article/10.3390/microorganisms14081643/s1. Figure S1. The frequency histogram. The number of sequences (x-axis) per sample (y-axis) is shown. Figure S2. Quality score by length. The sequence base (x-axis) and quality score (y-axis) are shown. Sequence quality starts to decay around 200 bases. For more details, see Supplementary Table S3 (forward-seven-number-summaries.tsv). Figure S3: Core microbiota heatmap of 90% genus prevalence and 1% abundance in samples at time W0. Samples are shown in the columns, while genera are in the rows. Additional top color labels are based on sample metadata according to the groups (IBS, UC). Figure S4: Beta diversity calculated using Aitchison distance. A Permutational Multivariate Analyses of Variance (PERMANOVA) was run for the samples; there was no difference between times using the formula distance ~ time + diet (for UC: Pr(>F) = 0.65, F = 0.92, R2 = 0.037; for IBS: Pr(>F) = 0.52, F = 0.99, R2 = 0.22). Table S1. Differential abundance analysis of gut microbiota taxa. Table S2. Sequence count summary. Table S3. Sequence length summary. Table S4. ASV sample count summary. Table S5. ASV taxa count summary. Table S6. Baseline vs final anthropometric, body composition, hematological, and biochemical parameters by intervention.
