# Supplementary material for: Microbiota and Nutritional Changes Following a Low-FODMAP Diet in Patients with Ulcerative Colitis and Irritable Bowel Syndrome: A Randomized Controlled Trial
Source: Microorganisms. 2026 Jul 28;14(8):1643. doi: 10.3390/microorganisms14081643 (PMC13515254; doi:10.3390/microorganisms14081643)
Supplement: Supplementary file 1 [file microorganisms-14-01643-s001.zip › microorganisms-4371441-supplementary.pdf]

SUPPLEMENTARY MATERIAL

Figures

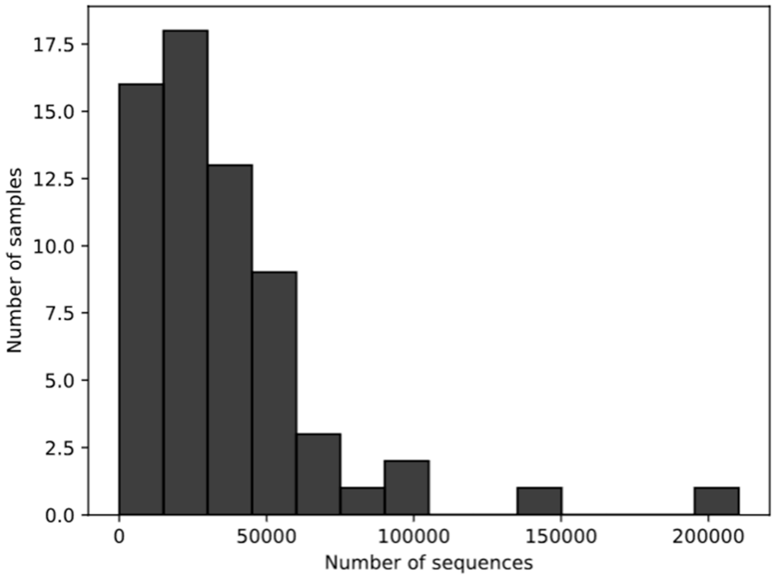

Figure S1. The frequency histogram. The number of sequences (x-axis) per sample (y-axis) is shown.

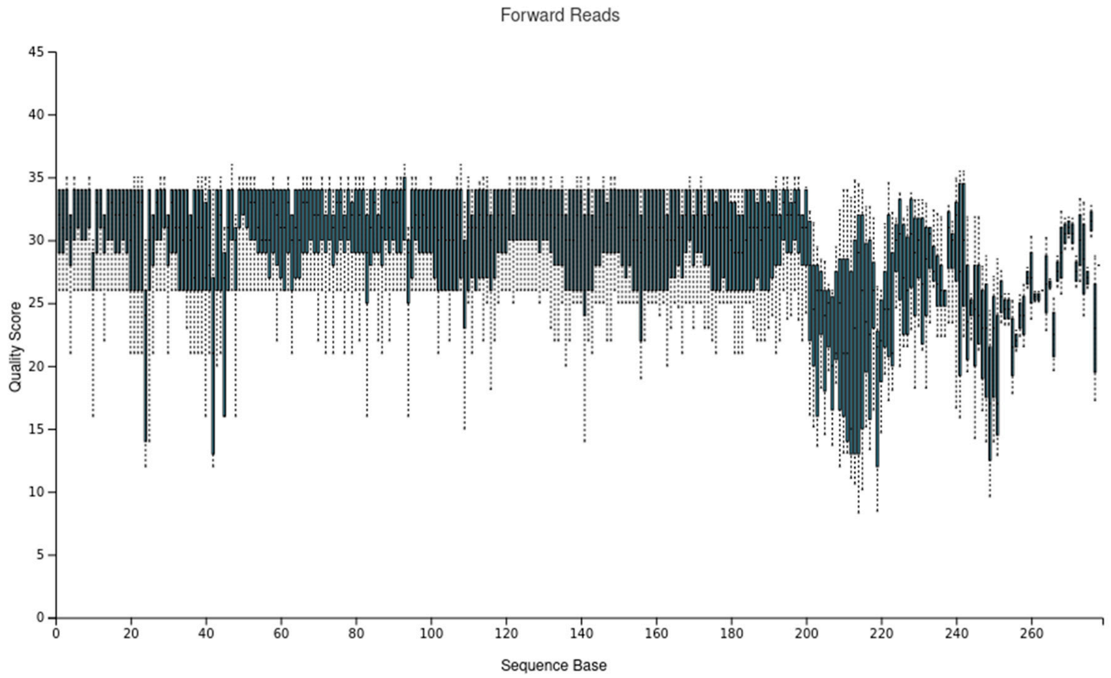

Figure S2. Quality score by length. The sequence base (x-axis) and quality score (y-axis) are shown. Sequence quality starts to decay around 200 bases. For more details, see Supplementary Table S3 (forward-seven-number-summaries.tsv).

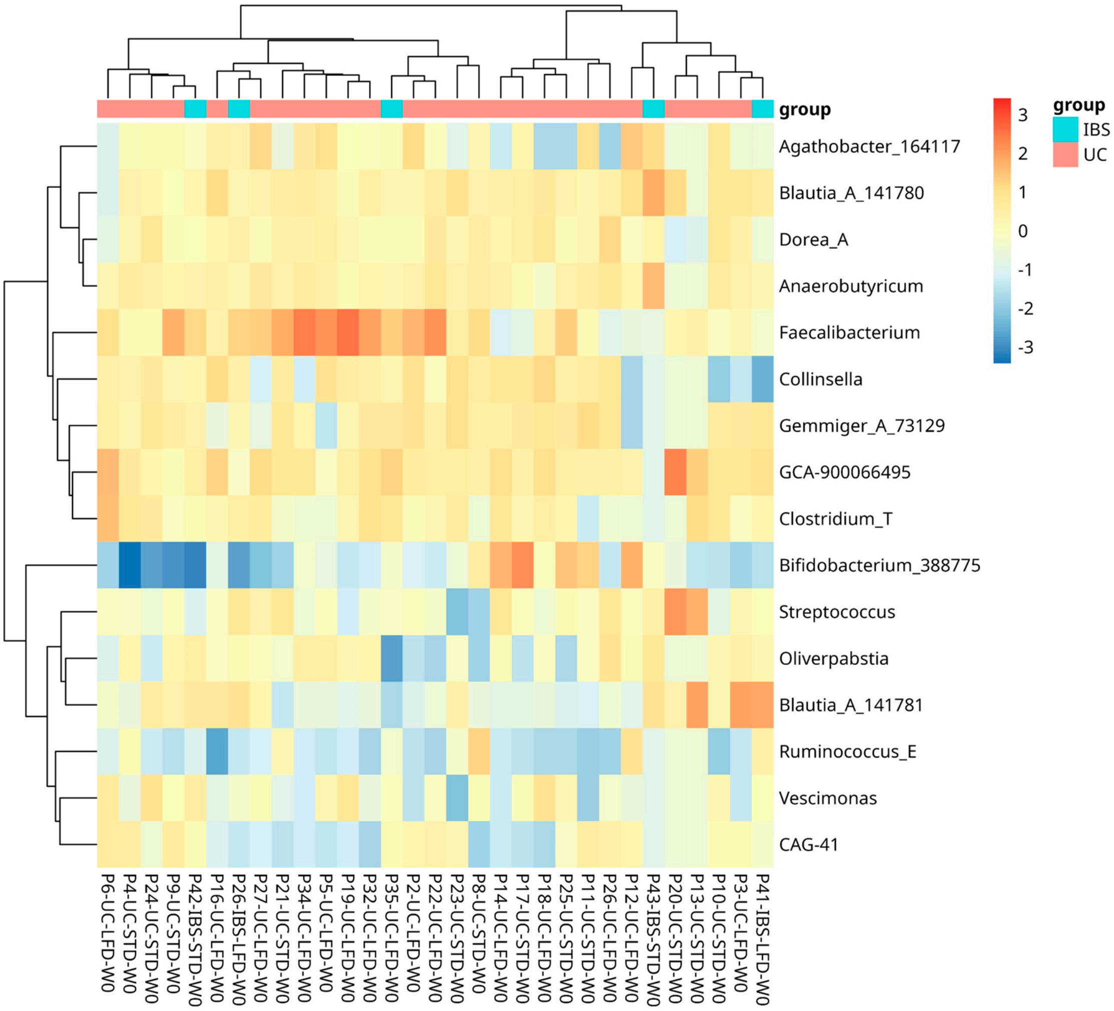

Figure S3: Core microbiota heatmap of 90% genus prevalence and 1% abundance in samples at time W0. Samples are shown in the columns, while genera are in the rows. Additional top color labels are based on sample metadata according to the groups (IBS, UC).

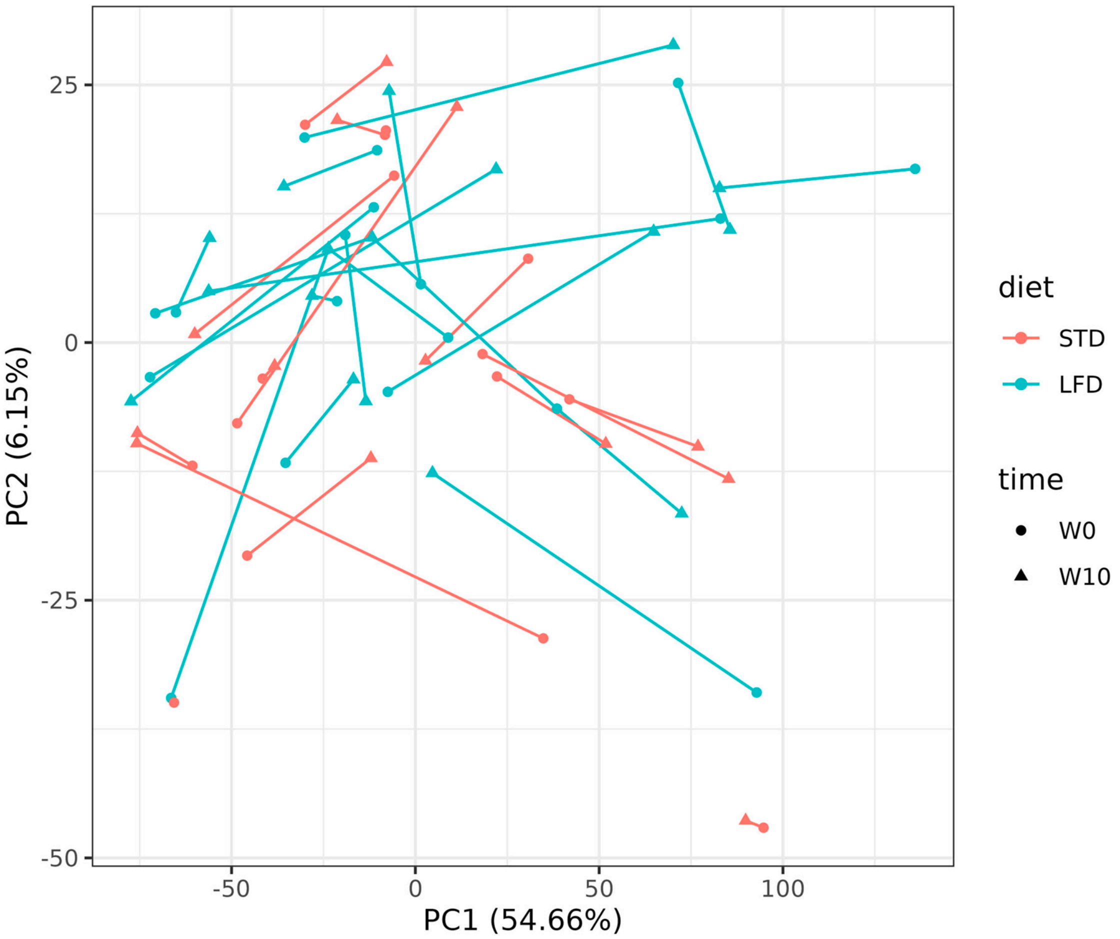

Figure S4: Beta diversity calculated using Aitchison distance. A Permutational Multivariate Analyses of Variance (PERMANOVA) was run for the samples; there was no difference between times using the formula distance ~ time + diet (for UC: Pr(>F) = 0.65, F = 0.92, R2 = 0.037; for IBS: Pr(>F) = 0.52 , F = 0.99 , R2 = 0.22). ‘

Tables

Table S1. Differential abundance analysis of gut microbiota taxa.

taxon	lfc_diet LFD	lfc_time W10	lfc_diet LFD: timeW10	q_diet LFD	q_time W10	q_diet LFD: timeW10
CAG-1427	-1.55	-1.37	2.75	1.56 e-05	1.00 e-02	0.03
CAG-177	2.76	1.41	-4.51	< 0.001	6.00 e-02	< 0.001
Evtepia	-0.25	-0.66	2.22	1.00	2.90 e-01	0.01
Angelakisella	2.00	1.79	-4.19	< 0.001	3.00 e-02	0.01
BX12	-1.38	-0.66	2.99	< 0.001	2.20 e-01	< 0.001
Holdemania	-0.27	-1.94	2.24	1.00	2.22 e-07	0.02
Granulicatella	-0.47	-1.82	2.19	1.00	4.02 e-05	0.03

The Log2 fold-change (lfc) values are shown for the diet assignment at W0 (lfc_dietLFD) for the effect of time (lfc_timeW10, in reference to the changes between W0 and W10), and their interaction (dietLFD: timeW10). The corresponding q-values (FDR-adjusted p-values) are reported for each taxon.

Table S2. Sequence count summary.

measure	value
Minimum	10.00
Median	28,605.50
Mean	35,660.66
Maximum	210,302.00
Total	2,282,282.00

Summary of sequence counts per sample of de-multiplexed data prior to dada2.

Table S3. Sequence length summary.

Percentile	length (nts)
2%	31
9%	57
25%	172
50%	173
75%	174
91%	180
98%	199

Distribution of sequence length (nts: nucleotides) in our samples. Percentages indicate the percent of samples bellow the length. A length of 172 was selected as a truncating parameter to keep approximately 75% of the reads.

Table S4. ASV sample count summary.

Measure	Before filtering	After filtering
Total	1,550,675.00	1,394,019.00
Minimum	3.00	1,507.00
First quartile	10,745.00	10,139.25
Median	20,652.00	17,622.50
Third quartile	31,477.50	29,236.25
Maximum	121,087.00	117,611.00

Summary of ASV counts in our samples. Measures are shown before and after filtering low-prevalence and low-abundance taxa.

Table S5. ASV taxa count summary.

Measure	Before filtering	After filtering
Total	1550675.00	1394019.00
Min	2.00	178.00
First quartile	13.00	463.75
Median	50.00	1483.00
Third quartile	191.00	3897.50
Max	162500.00	162500.00

Summary of ASV count per taxa. Measures are shown before and after filtering low-prevalence and low-abundance taxa.

Table S6. Baseline vs final anthropometric, body composition, hematological, and biochemical parameters by intervention.

	IBS n= 40						UC n= 34						Control n= 12					
	LFD n= 23			STD n= 17			LFD n= 15			STD n= 19			LFD n= 6			STD n= 6		
	**W0**	**W10**	*p*	**W0**	**W10**	*p*	**W0**	**W10**	*p*	**W0**	**W10**	*p*	**W0**	**W10**	*p*	**W0**	**W10**	*p*
Weight, kg	66.2 ± 8.1	66 ± 8.8	0.538	61.7 ± 8.8	62.6 ± 9.2	0.072	61.6 ± 10.8	62.4 ± 9.8	0.548	65.4±12.3	66.1±12.9	0.174	70.4±9.8	70.9±9.6	0363	74.1±12.3	75.1±13.5	0.175
BMI, kg/m²	26.9 ± 2.9	27.4 ± 3.1	0.124	**26 ± 3.8**	**26.4 ± 3.9**	**0.023**	24.1 ± 3.3	23.9 ± 3.1	0.609	25.2±3.5	25.4±3.7	0.190	24.7±3.5	24.9±3.4	0.378	26.3±2.1	26.6±2.4	0.200
Waist circumference, cm	84.5 ± 7.1	83.5 ± 6.9	0.125	81.7 ± 8.3	81.8 ± 8.4	0.882	79.9 ± 10.4	80 ± 10.1	0.129	82.4±10.7	82.6±11.5	0.757	80.9±10.2	80.3±9.6	0.584	82.2±8.1	81.5±8.3	0.659
Hip circumference, cm	101.1 ± 7.2	100.3 ± 7.4	0.248	97.7 ± 7.6	97.8±7.8	0.893	**99.1 ± 8.6**	**97.5 ± 8.3**	**0.040**	100.1±8.7	100.2±9.1	0.886	103.1±4.6	103.3±4.2	0.762	103.2±5.9	104.1±.4	0.064
Mid-upper arm circumference, cm	30.7 ± 2.8	30.8 ± 2.3	0.756	30.2 ± 3.8	30±3.1	0.528	29.8 ± 2.7	29.3 ± 3.1	0.105	31.2±4.2	31.1±4.4	0.774	29.8±2.2	29.1±2.4	0.083	32.1±2.6	31.9±3.2	0.745
Chest circumference, cm	97.9 ± 5.2	97.8 ± 5.8	0.838	97.1±7.8	97.7±7.7	0.057	93.1 ± 6.2	92.1 ± 6.4	0.156	96.4±8.5	96.1±8.9	0.570	92.7±7.1	92.1±7.2	0.284	97±4.1	97.8±5.1	0.197
PhA, grades	6.4 ± 1.06	6.4 ± 0.4	0.830	6.6±0.8	6.6±0.7	0.940	**6.1 ± 0.7**	**6.7 ± 1.1**	**0.026**	6.4±0.9	6.3±0.7	0.783	6.4±0.5	6.5±0.7	0.932	7.3±0.9	7.3±0.9	0.907
FM, kg	27.2 ± 6.1	26.6 ± 6.1	0.170	24.1±6.7	24.1±7.4	0.983	19.6 ± 6.9	18.1 ± 5.5	0.174	22.4±7.1	22.8±7.2	0.589	22.4±8.9	23.5±7.2	0.624	21.1±3.9	21.3±3.2	0.728
TBW, L	27.1 ± 3.1	27.4 ± 3.5	0.304	26.1±2.9	26.6±4.3	0.394	31.6 ± 7.1	32.2 ± 6.6	0.468	30.1±8.7	30.3±8.6	0.740	33.1±7.2	32.3±5.9	0.782	36.3±8.1	37±7.5	0.482
ICW, L	13.9 ± 1.6	14.1 ± 1.9	0.227	13.5±1.4	14.1±1.8	0.073	17.5 ± 4.8	18 ± 4.5	0.325	16.3±5.5	16.4±5.4	0.830	17.7±4.7	17.3±4.2	0.769	20.1±5.4	20.4±5	0.574
ECW, L	13.1 ± 1.5	13.2 ± 1.7	0.444	12.4±1.5	13.1±1.7	0.006	14.1 ± 2.3	14.2 ± 2.2	0.807	13.8±3.3	13.9±3.3	0.587	15.2±2.6	14.9±1.8	0.774	16.1±2.7	16.5±2.6	0.368
SMM, Kg	18.4 ± 3.1	18.5 ± 2.6	0.821	17.9±2.6	18.5±2.4	0.353	20.5 ± 4.8	21.9 ± 5.1	0.055	20.5±6.5	20.5±6.1	1.00	22.8±4.4	22.6±4.5	0.848	26.9±7.3	27.4±7.5	0.495
Leukocytes, x10e3/uL	6.9 ± 2.4	6.7 ± 1.5	0.547	6.3±1.8	6.8±2.1	0.291	7.4 ± 2.8	7.1 ± 1.8	0.244	7.1±2.8	6.5±2.3	0.320	5.7±1.3	5.4±0.9	0.439	7.7±1.7	7.3±1.5	0.387
Lymphocytes, %	28.7 ± 8.3	29.3 ± 7.5	0.510	30.5±7.7	27.2±5.8	0.055	30.6 ± 10.1	31.2 ± 10.9	0.748	28.8±9.5	27.1±7.3	0.436	32.7±5.3	33.1±5.9	0.880	30±8.8	30.1±9.6	0.982
Eosinophils, %	2.5 ± 1.9	2.4 ± 1.7	0.714	2.2±1.4	2.1±1.9	0.744	1.04 ± 1.07	2.5 ± 3.1	0.103	2.7±2.4	2.2 ± 1.8	0.204	**1.3±0.8**	**1.5±0.9**	**0.046**	1.2±0.5	1.4±0.2	0.254
Erythrocytes, x10 6/uL	4.8 ± 0.3	4.9 ± 0.4	0.177	4.7±0.2	4.7±0.2	0.621	4.8 ± 0.3	4.9 ± 0.4	0.512	4.9±0.3	4.8 ± 0.4	0.339	4.8±0.3	4.7±0.3	0.137	5.2±0.6	5.2±0.5	0.904
Hemoglobin, g/dL	14.5 ± 1.1	14.5 ± 1.1	0.784	14.6 ± 1.1	1.3±1.1	0.247	14.2 ± 2.1	14.5 ± 1.9	0.356	14.1±1.7	13.9±1.9	0.414	14.1 ± 1.6	13.8±1.5	0.091	16.1±1.6	16.2±1.5	0.757
Hematocrit, %	43.3 ±2.6	43.8 ± 2.4	0.140	43.4 ± 3.2	43.9±2.7	0.220	43.9 ± 5.3	44.8 ± 5.5	0.296	43.2±4.3	43.1±5.2	0.647	42.7 ± 4.5	42.2±4.4	0.324	49.1±4.9	48.9±4.5	0.844
MCV, fL	89.2 ± 4.9	89.3 ± 5.2	0.778	91.4 ± 3.6	92.8±3.4	0.024	89.9 ± 9.5	90.9 ± 9.2	0.311	87.6±9.1	88.3±8.8	0.300	88.3 ± 5.3	88.9±4.7	0.458	93.5±1.7	93.4±1.9	0.874
MCH, pg	29.8 ± 2	29.6 ± 2.2	0.371	30.4 ± 1.2	30.3±1.4	0.353	29.1 ± 3.8	29.4 ± 3.6	0.304	28.3±3.6	28.5±3.4	0.491	29.2 ± 2.2	29.1±1.7	0.814	30.7±0.6	31.1±0.7	0.125
Platelets, x10e3/uL	257.8 ± 83.1	257.1 ± 68.3	0.909	304.1 ± 94.9	306.7±83.1	0.714	333.1 ± 196.2	314.8 ± 166.9	0.154	322.5±172.6	319±158.8	0.799	317.6 ± 59.3	308.3±67.4	0.289	271.1±63.2	278.8±46.8	0.595
Iron, µg/dL	78.3 ± 19.5	80.8 ± 35.6	0.715	98.7 ± 56.7	78.4±26.2	0.168	88.8 ± 35.3	98.6 ± 46.9	0.535	70.1±34.2	85.4±48.1	0.095	95 ± 46.7	91.3±45.8	0.840	124.5±53.3	128.8±30.7	0.873
Glucose, mg/dL	94.5 ±13.1	97.4 ± 30.8	0.486	94.6 ± 10.7	93.7±11.1	0.610	98.7 ± 36.1	90 ± 15.2	0.195	93.8±6.5	93.6±11.4	0.934	101 ± 20.1	91±10.6	0.120	91.6±6.1	92±10.7	0.936

Urea, mg/dL	25.4 ± 7.9	29.1 ± 7.7	0.001	27.9 ± 6.4	25.4±6.7	0.176	27.2 ± 10.3	27.1 ± 10.1	0.972	28.9±6.2	31.7±8.2	0.163	26.8±6.6	34.8±8.4	0.005	25.5±6.3	28±8.2	0.450
Creatinine, mg/dL	0.6 ± 0.1	0.6 ± 0.1	0.692	0.7 ± 0.1	0.7±0.1	0.880	0.8 ± 0.1	0.8 ± 0.1	0.751	0.8±0.1	0.8±0.1	0.679	0.8±0.1	0.7±0.1	0.467	0.8±0.1	0.8±0.1	0.101
Total Cholesterol, mg/dL	180.7 ± 30.9	186.9 ± 28.6	0.224	191.4 ± 40.2	195.1 ± 38.9	0.402	171.8 ± 35.5	161.3±28.1	0.127	177.2±32.6	171.8±40.8	0.340	162.6±16.1	166.6±13.3	0.341	163±22.9	163.3±26.1	0.931
Triglycerides, mg/dL	161.9 ± 94.5	181.2 ± 184.4	0.497	110.4 ± 67.04	116.65 ± 65.1	0.461	132.8 ± 149.8	103.5±56.1	0.390	123.2±49.8	118.1±54.8	0.630	97.8±68.8	103.6±57.6	0.568	106.5±53.9	112±43.1	0.674
HDL-c, mg/dL	46 ± 9.2	47.6 ± 10.4	0.192	54.7 ± 18.9	57.6 ± 20.8	0.073	49.6 ± 15.7	50.6±9.6	0.668	50.1±10.4	52.7±11.2	0.203	51.5±5.5	54.1±9.7	0.500	47.5±10.1	46±8.3	0.537
LDL-c, mg/dL	112.9 ± 25.4	116.9 ± 25.4	0.357	120 ± 39.1	125.1 ± 37.2	0.186	94.7±16.6	100.5±30.2	0.354	112.1±32.6	104.5±34.2	0.148	100.3±8.5	101.5±4.9	0.783	105.6±26.1	116±33.7	0.168
Albumin, g/dL	4.2 ± 0.2	4.2 ± 0.2	0.203	4.2 ± 0.2	4.2 ± 0.2	0.906	4.3±0.4	4.4±0.2	0.694	4.3±0.2	4.3±0.3	0.856	4.5±0.2	4.4±.3	0.511	4.6±0.2	4.5±0.2	0.196
Calcium, mg/dL	9.4 ± 0.3	9.5 ± 0.3	0.194	9.3 ± 0.3	9.2 ± 0.2	0.109	9.5±0.2	9.5±0.3	0.869	9.4±0.2	9.4±0.3	0.922	9.7±0.5	9.5±0.5	0.324	9.6±0.3	9.5±0.3	0.215
Insulin, U/dL	9.7 ± 3.9	9.5 ± 4.3	0.685	9.1 ± 5.1	8.1 ± 4.2	0.283	5.8±3.1	6.1±3.3	0.721	8.1±6.1	7.8±6.1	0.805	12.4±12.1	10.2±11.2	0.056	6.7±4.3	12±14.2	0.412
Vitamin B12,	577.1 ± 393.8	554.3 ± 423.4	0.658	675.1 ± 416	1569 ± 3833	0.334	1008±1986	487.6±332	0.313	484.9±584.1	471.7±599.4	0.626	396.8±263.6	364.8±194.1	0.502	372.1±126	448.5±177.4	0.203
ESR, mm/hr	-	-	-	-	-	-	14.01±16.1	10.4±14.2	0.133	15.8±16.8	17.6±19.8	0.672	-	-	-	-	-	-
CRP, mg/L	-	-	-	-	-	-	6.6±7.1	7.3±11.5	0.814	9.1±19.8	10.8±19.4	0.735	-	-	-	-	-	-

W0: baseline assessment at week 0; W10: Final assessment at week 10; IBS inflammatory bowel syndrome; UC: Ulcerative Colitis; LFD: low FODMAP diet; STD: standard diet BMI: Body Mass Index; PhA: Phase Angle; FM: Fat Mass; L: liter; TBW: Total Body Water; ICW: Intracellular Water; ECW: Extracellular Water; SMM: Skeletal Muscle Mass; MCV: Mean Corpuscular Volume; MCH: Mean Corpuscular Hemoglobin; HDL-c: High-Density Lipoprotein Cholesterol; LDL-c: Low-Density Lipoprotein Cholesterol; ESR: Erythrocyte Sedimentation Rate; CRP: C-Reactive Protein. All the results are presented as mean ± standard deviation. Paired t-test, significant p value <0.05.
